# Role of Genetic Alterations in the *NLRP3* and *CARD8* Genes in Health and Disease

**DOI:** 10.1155/2015/846782

**Published:** 2015-02-18

**Authors:** G. V. Paramel, A. Sirsjö, K. Fransén

**Affiliations:** Department of Clinical Medicine, School of Health and Medical Sciences, Örebro University, 701 82 Örebro, Sweden

## Abstract

The complexity of a common inflammatory disease is influenced by multiple genetic and environmental factors contributing to the susceptibility of disease. Studies have reported that these exogenous and endogenous components may perturb the balance of innate immune response by activating the NLRP3 inflammasome. The multimeric NLRP3 complex results in the caspase-1 activation and the release of potent inflammatory cytokines, like IL-1*β*. Several studies have been performed on the association of the genetic alterations in genes encoding NLRP3 and CARD8 with the complex diseases with inflammatory background, like inflammatory bowel disease, cardiovascular diseases, rheumatoid arthritis, and type 1 diabetes. The aim of the present review is therefore to summarize the literature regarding genetic alterations in these genes and their association with health and disease.

## 1. Introduction

In the beginning of the 2000s, mutations in genes encoding the NLRP3 inflammasome components were associated with a spectrum of diseases with profound systemic inflammatory state [1, 2]. Since its discovery, the genetic alterations in the genes encoding the NLRP3 inflammasome have increasingly attracted the research interest, with respect to several diseases with an inflammatory background as a possible predisposing factor in diseases like inflammatory bowel disease (IBD) [3, 4], rheumatoid arthritis (RA) [5, 6], Alzheimer's disease (AD) [7], type 1 diabetes (T1D) [8], and atherosclerosis [9, 10].

The purpose of this review is to summarize and discuss the association of genetic variants in genes encoding proteins that have been associated with the NLRP3 inflammasome and the risk of inflammatory diseases. To date, numerous genome-wide association studies (GWAS) have been performed with respect to various diseases and have uncovered several genetic susceptibility loci associated with increased disease risk. However, the SNPs identified with GWAS technique probably account for a fraction of the hereditary factors, since the remaining heritability lies in genetic alterations that do not achieve genome-wide significance, possibly due to incomplete linkage disequilibrium between the uncovered variants and casual variants with lower minor allele frequency [11]. Therefore, the present review is focused on the association of genetic alterations in the* NLRP3* and* CARD8* genes to inflammatory diseases, identified with a candidate gene approach.

## 2. Activation of the NLRP3 Inflammasome

The NLRP3 inflammasome consists of NLRP3 scaffold protein, ASC (PYCARD) adaptor protein, and caspase-1 protein. The Caspase recruitment domain-containing protein 8 (CARD8; also known as TUCAN/CARDINAL) has in some studies also been associated with the NLRP3 complex, although its role for inflammasome activation is not completely clear [12]. Initially, CARD8 was shown to be a regulator of NF-*κ*B and caspase-1 activation and NOD-2 signaling [13–16]. Though recent studies have shown that CARD8 negatively regulates NLRP3, studies have also shown that CARD8 has no role in the IL-1*β* release [17, 18].

The NLRP3 inflammasome can be activated by several different factors, such as pathogen associated molecular patterns (PAMPs) like bacterial lipopolysaccharide and different microorganisms (*C. albicans, S. cerevisiae, L. monocytogenes, S. aureus, *and* P. gingivalis*) and viruses (adenovirus, Sendai virus, andinfluenza virus). Furthermore, damage associated molecular patterns (DAMPs) like extracellular ATP, uric acid, elevated glucose levels, cholesterol crystals, calcium pyrophosphate dehydrate (CPPD), monosodium urate, and different pollutants (silica, asbestos, UV radiation, and skin irritants) have also been implicated as NLRP3 inflammasome activators [19]. Upon NLRP3 activation, the homotypic interaction of PYD of NLRP3 and ASC proteins leads to autocleavage of procaspase-1 to active caspase-1 followed by the processing of the inactive proinflammatory cytokines IL-1*β*, IL-18, and IL-33 to their active forms [12, 20, 21]. The mechanism involved in the assembly and formation of NLRP3 inflammasome protein complex for the downstream processing of IL-1*β* includes lysosomal destabilization due to phagocytosed particulate and crystalline moiety, mitochondrial damage due to intracellular K^+^ efflux and Ca^2+^ mobilization, and ROS induction by mitochondria and NLRP3 activators [22–30].

Studies have found certain proteins of endogenous origin that may possibly negatively regulate the NLRP3 inflammasome in host, serving as checkpoints to regulate the immune responses. TRIM family proteins, nitric oxide, microRNA, IFNs, CD40 ligands, and autophagy are the known endogenous regulators to the NLRP3 inflammasome [31–38]. Certain proteins of microbial origin are also found to regulate the NLRP3 inflammasome such as viral pyrin (PYD) and V protein of measles virus [39, 40]. These studies suggest that many agents may contribute to the regulation of NLRP3 inflammasome activation to prevent the overexpression and hyperactivity of the inflammasome due to an accidental trigger.

## 3. Cryopyrin Associated Periodic Syndrome (CAPS) and Genetic Alterations in the* NLRP3* Gene

The discovery in 2001 by Hoffman and colleagues on genetic alterations in the* NLRP3* gene as a prime source for the set of autosomal dominantly inherited inflammatory diseases, combinedly known as cryopyrin associated periodic syndrome (CAPS), was a breakthrough in the clinical execution of these diseases [1]. CAPS is a family of autosomal dominantly inherited diseases like familial cold autoinflammatory syndrome (FCAS), Muckle-Wells syndrome (MWS), and chronic infantile neurological cutaneous articular syndrome (CINCA), also known as neonatal onset multiple inflammatory syndrome (NOMID), sharing overlapping characteristic clinical symptoms of recurrent fever, increased white blood cell count, and inflammation. The etiology of CAPS is mainly contributed to the gain of function mutations in the* NLRP3* gene, which leads to upregulated amounts of IL-1*β*. Around 60% of CAPS patients carry an activating mutation in the* NLRP3* gene [41, 42]. This however does not exclude that the additional CAPS cases also might be carriers of mutations in the* NLRP3* gene. Tanaka and coworkers found that approximately 70% of the CINCA/NOMID cases without* NLRP3* mutation detected by sequencing were subjects to somatic mosaicism [42].

The majority of the* NLRP3* mutations in CAPS are missense mutations located in exon 3 of the* NLRP3* gene (http://fmf.igh.cnrs.fr/ISSAID/infevers/) which encodes for the NACHT domain, responsible for autorepression of NLRP3 oligomerization by interacting with the LRR domain in healthy cellular condition, and is also involved in the interaction with CARD8 [12, 29]. In addition to germline mutations in the CAPS diseases, several single nucleotide polymorphisms (SNPs) in the genes encoding the NLRP3 inflammasome components or in their regulatory regions have been associated with the pathophysiology of various other diseases. The polymorphisms discussed in the present paper are SNPs located in the* NLRP3* and* CARD8* genes and in putative regulatory regions.

## 4. Functional Aspects on the Q705K and the C10X Polymorphisms in the* NLRP3* and* CARD8* Genes

**Figure 1:**
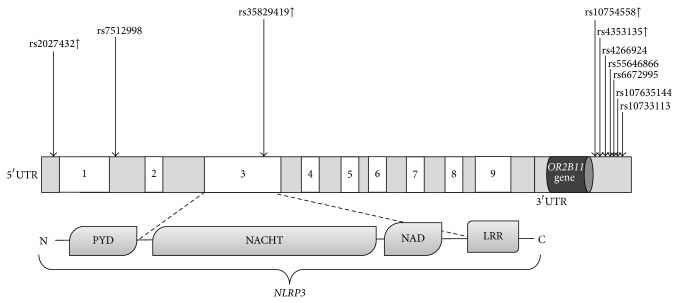
Schematic representation of polymorphisms in the* NLRP3* gene investigated in relation to inflammatory diseases. Exons of the* NLRP3* gene are displayed as white boxes (not to scale). Upregulation of NLRP3 is indicated as (**↑**) beside the SNP rs number; polymorphisms with unknown biological function are not labeled. The lower panel represents the different domains of NLRP3: PYD, pyrin domain; NAD, NACHT associated domain; and LRR, leucine-rich repeat.

The Q705K polymorphism (previously known as Q703K in the Infevers database located in exon 3, rs35829419) of the* NLRP3* gene is a low penetrant variant associated with atypical CAPS features and with several other inflammatory diseases. The Q705K variant is a gain of function alteration leading to excessive IL-1*β* and IL-18 production (Figure 1) [43].

**Figure 2:**
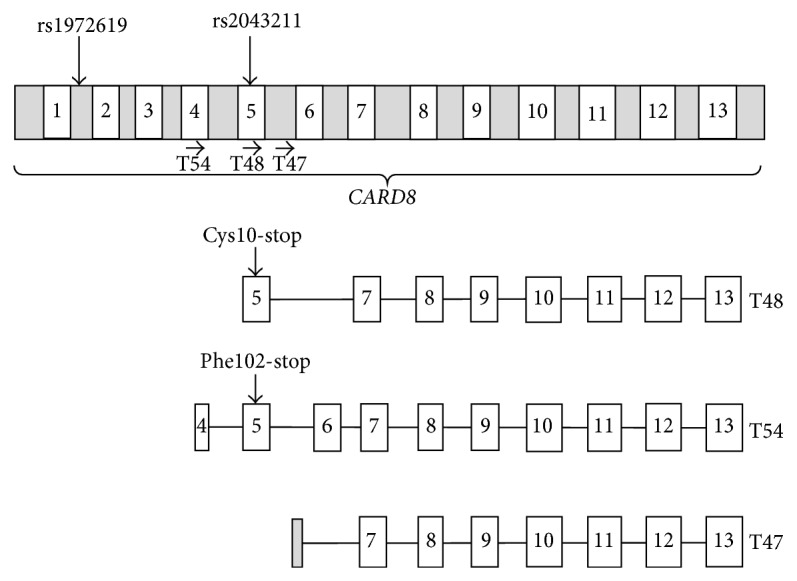
Schematic representation of polymorphism in the* CARD8* gene and mRNA isoforms (modified from Bagnall et al. 2008 [45]; not to scale). Exons of the* CARD8* gene are displayed as white boxes. The arrow (**→**) represents the site of open reading frame (ORF) for the given isoforms.

Another polymorphism extensively studied is the C10X polymorphism in the* CARD8* gene (rs 2043211; Figure 2) in relation to several different diseases, but the variant alone has not been associated with the CAPS disease. The C10X of the* CARD8* gene is a nonsense mutation located in exon 5 of the* CARD8 *gene leading to a truncated CARD8 protein. Studies have shown that the polymorphic allele was associated with elevated cell death* in vitro*, although the actual role of CARD8 is not clear [44]. Functional investigation of* CARD8* for inflammasome assembly is complicated by the fact that* CARD8* is missing in rodents. Studies by Bagnall and coworkers have revealed a series of isoforms that can possibly affect the functional consequences of the C10X (rs2043211) polymorphism [45]. The functional consequence of C10X variants on the two known isoforms of CARD8, T48 and T54, generates a Cys>Stop at codon 10 in T48 and Phe>Ile amino acid substitution at codon 52 in T54. The discovery of additional three novel mRNA isoforms of* CARD8*, T47, T51, and T60, might explain the different functional consequences of C10X variant. In fact, the high rate of homozygous patients with loss of function polymorphism for what appears to be human knock-outs might reflect partial rescue of CARD8 function by alternative splicing, leading to an almost functional full length protein. For instance, the transcription of T47 begins downstream of the C10X, thereby not affecting the isoform (Figure 2). Patients homozygous for the C10X genotype might therefore have a functional CARD8 protein due to the T47 isoform [44]. A detailed analysis determining the underling effect of the functional variants of CARD8 remains to be investigated.

The review focuses mainly on these variants, since several studies have reported that Q705K and C10X variant* per se* or in combination are associated with increased risk of chronic inflammation. An overview of all of the genetic localizations of the SNPs of* NLRP3* and* CARD8* genes covered in the present review can be found in Table 1 and Figures 1 and 2, and their potential functionality in relation to disease will be described in each section below.

## 5. Role of SNPs in* NLRP3* and* CARD8* Genes in Blood Donors

The functional characterization of the SNPs in the complex disease is challenging with enormous environmental and genetic factor exerting deleterious effect on the disease pathophysiology. However, functional studies of SNPs performed on the genotyped healthy individuals might overcome the effect attributed by disease severity and therapy, thereby providing insight to the functionality of SNP in health.

In a recent study, carriers of a combination of the polymorphisms Q705K (rs35829419) and C10X (rs2043211) in the* NLRP3* and* CARD8 *genes, respectively, were associated with elevated levels of IL-1*β* and IL-33 in plasma, when compared to noncarrier controls [46]. This indicates that the genetic variants in NLRP3 inflammasome related genes may influence the threshold of inflammasome activation and perturb the balanced immune response, thereby suggesting the role of these variants in influencing the basal active state of innate immune response.

## 6. Role of SNPs in* NLRP3* and* CARD8* Genes in Diseases with Inflammatory Background

### 6.1. Inflammatory Bowel Disease

Inflammatory bowel disease (IBD), comprising to Crohn's disease (CD) and ulcerative colitis (UC), is a multigenic complex disease of dysregulated mucosal immune response of gastrointestinal tract to the commensals of gut flora in the genetically susceptible host [63]. In recent years, the genetics of IBD has been revolutionized by the identification of increased number of IBD susceptibility loci, thereby providing novel insight in the pathophysiology and treatment options of a complex genetic disease. In addition to the* NOD2* locus, the* NLRP3* locus is one amongst the several newly discovered CD loci conferring the genetic susceptibility to IBD [47, 64].

To date, several SNPs in the* NLRP3* region have been genotyped to study the association with CD. The rs4353135, rs4266924, rs55646866, rs6672995, rs107635144, and rs10733113 SNPs in a regulatory region downstream the* NLRP3* gene (Figure 1, Table 1) were found significantly associated with CD in five European cohorts [47]. Although the SNPs were strongly associated with the risk of CD, the association with* NLRP3* expression and IL-1*β* production was conferred only by rs4353135 and rs6672995, respectively. The risk alleles of rs4353135 and rs6672995 were found to be associated with lower* NLRP3* and IL-1*β* expression, respectively, in the peripheral blood of healthy donors [47]. On the contrary, no significant association was found between the variants and CD in a different sample set of CD patients from UK [65]. The conflicting results due to the lack of replication should therefore be interpreted cautiously.

In addition, it was found that IBD patients homozygous for the rs2043211 SNP encoding the C10X polymorphism in the* CARD8* gene (Figure 2) retained immunoreactive isoform of CARD8, thereby revealing the necessity of detailed characterization of disease associated variants to the contribution to the disease and geographical differences influencing the genetic background of the IBD patients. In a Korean cohort, the rs2043211 SNP was found to be significantly associated with UC. Moreover, the stop allele of rs2043211 was associated with increased IL-1*β* level in serum of UC patients [48]. Conflicting results have also been reported on the association of rs2043211 with CD and UC. Significant association of rs2043211 was reported with CD in a British cohort [49], although other studies showed no evidence of such association [66]. The conflicting results might hypothetically be due to the interaction of rs2043211 with the gain of function mutation rs35829419 in the* NLRP3* gene, in a similar fashion as described above for healthy individuals, but this needs to be confirmed. This idea is supported by the fact that the combined polymorphisms of* CARD8* and* NLRP3* (rs2043211/rs35829419) were shown to be associated with CD, indicating a possible interaction between these variants in the pathogenesis of CD in two separate studies [50, 51]. In addition, a* CARD8* SNP from intron 1, rs1972619 (Figure 2), was also found to be significantly associated with CD in Koreans [48]. However, the functional role of the rs1972619 is yet to be discovered. Altogether, these findings reflect that the dysregulation of NLRP3 inflammasome might have a prominent role in the pathogenesis of IBD.

### 6.2. Cardiovascular Diseases

In the past decade, the mortality rate of cardiovascular diseases has declined; however it remains to be the leading cause of death worldwide [67, 68]. Increasing evidence suggests that inflammation in the coronary atherosclerosis leading to sudden alteration of the plaque stability often accelerates ischemic cardiovascular events such as myocardial infarction [69]. Extensive clinical and pathophysiological research confirms that therapeutic intervention targeted against inflammatory mediators is effective for the treatment of myocardial infarction [70, 71]. Among the vast number of inflammatory cytokines, IL-1*β* is known to be a key modulator in the complex vessel wall inflammation and acceleration of atherogenesis process [72, 73]. Blockade of IL-1*β* prevented the adverse cardiac remodelling and lowered the incidence of heart failure after myocardial infarction [74, 75]. Also, NLRP3^−/−^ and ASC^−/−^ animal models have shown the role of NLRP3 in the progression of atherosclerosis and myocardial dysfunction, although it has been questioned [9, 10, 76, 77].

Association studies have found that the Q705K polymorphism in the* NLRP3* gene (rs35829419; Figure 1) conferred a protective effect against the risk of developing MI in females and was also associated with increased CRP levels in males [52]. Also in another study the SNP rs35829419 revealed a significant association with the increased IL-1*β* levels and showed a trend to the lower levels of CRP in plasma [54].

Regarding the variants of the* CARD8* gene, the C10X variant (rs2043211; Figure 2) was found to be associated with lower expression of* CARD8* in plaque as well as with the lower levels of CRP and MCP-1 in plasma, but the SNP showed no association with the risk of myocardial infarction in Swedish cohorts [78]. However, in a Chinese cohort, the rs2043211 was associated with ischemic stroke [53]. A study from Spanish cohort also showed that rs2043211 was not associated with risk of developing cardiovascular events in RA patients [79]. Moreover, the interaction between the Q705K* NLRP3* polymorphism and C10X polymorphism in the* CARD8 *gene (rs35829419/rs2043211, CC/AT versus CC/AA) conferred a modest protective effect against abdominal aortic aneurysms (AAA) [54].

Collectively, taking these genetic findings into account, it is likely that these variants might modulate the basal active state of immune response and might thereby contribute to the pathophysiology of cardiovascular diseases.

### 6.3. Rheumatoid Arthritis

The pathogenesis of rheumatoid arthritis (RA) is driven by a chronic inflammation of the joints that leads to the irreversible destruction of the joints. Among the several cytokines involved in the pathogenesis of RA, IL-1*β* is one of the prominent cytokines released from monocytes to induce and perpetuate the chronic inflammatory process in the joints [80]. The effective blockade of IL-1*β* and the inhibition of NLRP3 inflammasome to block IL-1*β* and IL-18 response are the ongoing therapeutic targets for the treatment of RA [5, 81, 82]. However, in mice models of RA, the NLRP3 inflammasome was found to play a limited role in the pathophysiology of RA and the activation of IL-1*β* was independent of NLRP3 activation [83]. Since the animal model differs from RA in human, further clinical studies are required to confirm the role of NLRP3 in RA.

The genetic background of NLRP3 inflammasome in the pathogenesis of RA is complex and unclear. The Q705K polymorphism in the* NLRP3* gene (rs35829419; Figure 1) was not associated with the RA susceptibility [6]. However, the C10X variant (rs2043211; Figure 2) of* CARD8 *was found to affect the 28-joint disease activity score, erythrocyte sedimentation rate, and tender joint count in patients [56]. In addition, the rs4353135 polymorphism (G variant; Figure 1) located in the* NLRP3* regulatory region was found to be associated with risk of oligoarticular/polyarticular juvenile idiopathic arthritis [55]. The variant was also associated with increased inflammatory marker such as CRP, ESR, and lymphocyte IL-17 levels [55]. Though the epistatic effect of combined SNP in* NLRP3* and* CARD8* (Q705K, rs35829419 and C10X, rs2043211) was found to be associated with the RA susceptibility and severity, the replication of the results was not observed in the prospective study from the same research group [6, 56]. However, the functional studies have shown that the combined polymorphism is associated with increased caspase-1 activity and IL-1*β* levels [84]. Taken together, the genetic variants of NLRP3 inflammasome are likely to influence the RA disease progression, but, with few mechanistic human studies, it is too early to confirm the profound role of these variants in the RA disease pathology.

### 6.4. Ankylosing Spondylitis

Ankylosing spondylitis (AS) is a chronic inflammatory disease of spinal and sacroiliac joints, caused by bone and joint erosion [85]. Also, the status of IBD is highly prevalent in the AS patient. Genetic alterations have been implicated as potent factor in the etiology of the disease. Among the different genes associated with the AS susceptibility, IL-1 gene family has also been proposed in the susceptibility of AS in Europeans [86].

To our knowledge, the study made by Kastbom et al. is the only investigation conducted on the association of genetic alteration in the NLRP3 inflammasome components with the AS susceptibility. SNPs in the* NLRP3* region were not associated with the risk of AS in the Swedish population; however the C10X (rs2043211; Figure 2) polymorphism of the* CARD8* gene was found to be associated with decreased risk of AS in dominant model [57]. Further functional studies and replication of association in independent cohorts are required to establish the mechanism behind the protective effect conferred by the C10X variant against AS.

### 6.5. Celiac Disease

Celiac disease is an inflammatory disease in genetically predisposed individuals characterized by the destruction of small intestinal lining in response to dietary protein present in wheat, barley, and rye. Celiac disease is often found in conjunction with other autoinflammatory diseases and the disease shares several immunological features with IBD [87]. In a case report, celiac disease was found in patients with cryopyrinopathies suggesting the likelihood of excessive IL-1*β* production in the generation of reactive T cells that can promote the pathophysiology of celiac disease [88]. The unique celiac disease diagnostics is based on circulating transglutaminase autoantibodies in combination with biopsy. Presence of the genetic variation of HLA-DQ2 and HLA-DQ8 is in strong association with celiac disease [89].

Among the several NLRP3 inflammasome associated SNPs investigated, in association with celiac disease, the rs35829419 (Q705K; Figure 1) polymorphism in the* NLRP3* gene was found significantly associated with celiac disease in two independent studies of Italian and Brazilian origin and conferred a protective effect [58, 59]. However, a significant amount of research remains to be performed on the genetic and functional aspects of NLRP3 inflammasome variants in the predisposition to the risk of celiac disease.

### 6.6. Type 1 Diabetes

Progressive destruction of insulin secreting islets cells of pancreas due to autoimmune response leads to type 1 diabetes (T1D). The major complication of the disease resides overtime in its effect on different organs. Cardiovascular diseases, neuropathy, kidney disease, lower extremity amputation, and blindness are some of the serious consequences of T1D [90]. In addition to the insulin replacement therapy, the recent advancement in the therapeutic targets also includes a multicentre randomized clinical trial to examine the effect of IL-1 antagonism on beta cells in T1D, since IL-1*β* was found to induce apoptosis of pancreatic beta cells [91, 92]. Among the genetic factors, HLA-DQ beta gene is strongly associated with T1D [93]. Also studies performed in NLRP3^−/−^ animal models have shown improved insulin sensitivity and glucose tolerance [8]. The combination of immunological and genetic features is useful in the prediction of the disease.

In a Brazilian cohort, the Q705K gain of function variant (rs35829419; Figure 1) in the* NLRP3* gene was not associated with the risk of T1D [58]. To our knowledge, no association studies have been published on the C10X variant (rs2043211; Figure 2) in the* CARD8* gene and T1D. However, the SNP rs10754558 in the downstream regulatory region (Figure 1) of* NLRP3* was found to be significantly associated with T1D in patients of Brazilian origin and exerted a protective effect on the disease [58]. Functional studies have shown that rs10754558 was associated with* NLRP3* mRNA stability, since the G allele increases the* NLRP3* expression 1.3-fold when compared to C allele [94]. These results indicate that the protection to the disease exerted by the variant possibly depends on the stable expression of the* NLRP3*. As T1D influences the development of several inflammatory diseases, it is likely that the NLRP3 inflammasome variants associated with several diseases might have a profound effect on the pathophysiology of diabetes. With very few studies performed to date, it is therefore difficult to draw any general conclusion regarding the role of NLRP3 inflammasome variants in the development of T1D.

### 6.7. Gout

Gout is the most common form of autoinflammatory arthritis and is characterized by the intra-articular deposition of the monosodium urate (MSU) and elevated serum urate. To date, there are very few studies conducted on the association of* NLRP3* polymorphisms and the risk of gouty arthritis. In a recent study, the intronic rs7512998 (Figure 1) located in the* NLRP3* gene showed a significant difference in the genetic frequency among the 17 tag SNPs from the* NLRP3* gene that were investigated in association with the susceptibility to gouty arthritis in 480 primary gout and control patients [60]. However, other potential susceptible variants remain to be investigated in association with the risk of gouty arthritis and functional influence of the variant on the disease pathology. Given that the DAMPs are well-known direct activators of NLRP3 inflammasome and IL-1*β* production, further studies on variants responsible for the dysregulation of the NLRP3 inflammasome may provide an insight on the susceptibility of developing gout.

### 6.8. Alzheimer's Disease

Alzheimer's disease (AD) is a neurodegenerative disease characterised by chronic deposition of amyloid *β* and is the most common form of dementia in the elderly [95, 96]. Studies have shown that the formation of amyloid *β* plaques is associated with the activation of NLRP3 inflammasome and IL-1*β* release in microglia, thereby enhancing the AD progression by aggravating inflammatory response [97]. Since NLRP3^−/−^ and caspase-1^−/−^ animal models have confirmed enhanced amyloid *β* clearance and protection from the loss of spatial memory [97], blocking of NLRP3 activation and the NLRP3 inflammasome derived cytokines might be a new therapeutic intervention for AD.

A recent study analyzing 1133 late-onset AD (LOAD) patients and 1159 healthy controls found that genetic variants, Q705K (rs35829419), rs10754558, and rs2027432, in or in association with the* NLRP3* gene (Figure 1) were associated with the risk of LOAD in Northern Han Chinese [61]. Among the* NLRP3* polymorphisms, the rs35829419 (Q705K in* NLRP3*) was found to confer protection against the risk of AD [61]. The association conferring the protective effect is consistent with our report on the risk of MI [52]. In another study, the* CARD8* variant rs2043211 (C10X; Figure 2) was found to be associated with increased risk of developing AD in women [62]. The rs10754558 variant of the* NLRP3* gene was also strongly associated with AD in ApoE *ε*4 carriers and appears to interact with ApoE gene to impart a synergistic effect on the risk of AD [61]. Moreover, the rs2027432 variant of the 5′ flanking region of* NLRP3* gene (Figure 1) was found to be strongly associated with risk of LOAD and studies support that the variant might lead to increased expression of* NLRP3* by enhancing the transcriptional activity of* NLRP3* promoter [61, 98]. Collectively, it seems that the different variants of* NLRP3* gene have a potential effect to the disease severity, but further studies are warranted to confirm and explore the role of NLRP3 variants in the susceptibility of AD.

## 7. Concluding Remarks

In recent years, several studies have confirmed the potential role of the NLRP3 inflammasome in the pathophysiology of inflammatory diseases. The results from the genetic studies also support the role of variants from NLRP3 inflammasome associated genes as susceptible candidates for different inflammatory diseases by influencing the balance of immune response. The functionally well-known Q705K polymorphism (rs35829419) in the* NLRP3* gene was found to confer protective effect on AD, celiac disease, and female myocardial infarction but not on T1D or RA. Regarding the C10X polymorphism in the* CARD8* gene, conflicting association outcomes are obtained for rs2043211 to the different disease susceptibility. The combinatory effect of the SNPs in the* NLRP3* and* CARD8* genes did also show a conflicting outcome amongst the different diseases. Several other SNPs are found to exert deleterious effect on the susceptibility of diseases; however further studies are required to replicate the association in order to confirm the role of these variants in the respective diseases. Further functional studies elucidating the role of identified SNPs on the transcriptional and protein level of the gene in different disease models will lead to the understanding of NLRP3 inflammasome in the etiology of inflammatory diseases. The primary goal of such genetic finding will contribute to the prediction, prevention, and development of new common diagnostics and therapeutic strategies in the treatment of autoimmune and inflammation related disorders.

## Figures and Tables

**Table 1:** Association studies confirming the role of *NLRP3* and *CARD8* gene polymorphisms in inflammatory disease.

Disease	Gene	SNPs	Cohorts	Reference
CD	*NLRP3 *	rs4353135, rs4266924, rs6672995, rs10733113, rs107635144, rs55646866	European	[47]
	*CARD8 *	rs2043211	Korean, British	[48, 49]
		rs1972619	Korean	[48]
	*NLRP3/CARD8 *	rs35829419/rs2043211	New Zealander, Swedish	[50, 51]

CVD	*NLRP3 *	rs35829419	Swedish (MI)	[52]
	*CARD8 *	rs2043211	Chinese (ischemic stroke)	[53]
	*NLRP3/CARD8 *	rs35829419/rs2043211	New Zealander (AAA)	[54]

RA	*NLRP3 *	rs4353135	Taiwanese	[55]
	*CARD8 *	rs2043211	Swedish	[56]
	*NLRP3/CARD8 *	rs35829419/rs2043211	Swedish	[57]

AS	*CARD8 *	rs2043211	Swedish	[57]

Celiac disease	*NLRP3 *	rs35829419	Italian and Brazilian	[58, 59]

T1D	*NLRP3 *	rs10754558	Brazilian	[58]

Gout	*NLRP3 *	rs7512998	Chinese	[60]

AD	*NLRP3 *	rs35829419	Northern Chinese	[61]
		rs10754558	Northern Chinese	[61]
		rs2027432	Northern Chinese	[61]
	*CARD8 *	rs2043211	Brazilian	[62]

CD: Crohn's disease; CVD: Cardiovascular diseases; RA: Rheumatoid arthritis; AS: Ankylosing spondylitis; T1D: Type 1 diabetes; AD: Alzheimer's disease; MI: Myocardial infarction; AAA: Abdominal aortic aneurysms.
